# In-Vivo Evaluation of an In Situ Polymer Precipitation Delivery System for a Novel Natriuretic Peptide

**DOI:** 10.1371/journal.pone.0052484

**Published:** 2013-02-18

**Authors:** Soo Ghim Lim, Subbu S. Venkatraman, John C. Burnett, Horng H. Chen

**Affiliations:** 1 School of Materials Science and Engineering, Nanyang Technological University, Singapore, Singapore; 2 Cardiorenal Research Laboratory, Division of Cardiovascular Diseases, Mayo Clinic, Rochester, Minnesota, United States of America; University of Minho, Portugal

## Abstract

This study reports on the release of a novel natriuretic peptide, CD-NP, from an in situ polymer precipitation delivery system. Following extensive screening of in-vitro release profiles, an in-vivo evaluation of the efficacy of the delivery system was carried out in Wistar rats. Gel injection was performed subcutaneously on the back of the rats. A secondary messenger, cyclic Guanosine 3′5′ Monophosphate (cGMP), was tested for verification of CD-NP bioactivity, in addition to direct measurements of CD-NP levels in plasma and urine using a radio-immuno assay. Plasma evaluation showed an elevated level of CD-NP over 3 weeks' duration. Unexpectedly, plasma cGMP level followed a decreasing trend over the same duration despite high CD-NP level. Loss of drug bioactivity was ruled out as a high level of CD-NP and cGMP excretion was observed in the treatment group as compared to baseline readings. This unexpected low-plasma cGMP levels and high-urinary cGMP excretion suggest that there might be other compensatory responses to regulation of the CDNP bioactivity as a result of the high drug dosing. The results stress the importance of assessing the overall bioactivity of released drug (in-vivo) concurrently in addition to measuring its concentrations, to determine the correct release profile.

## Introduction

Worldwide, cardiovascular diseases have emerged as the leading cause of death [1]. Various forms of cardiovascular diseases result in the development of the syndrome of heart failure (HF). HF is a condition in which the heart fails to supply sufficient blood to meet the needs of the body. Characteristics of HF include functional and structural changes in the heart, endothelial and vascular dysfunction with vasoconstriction, sodium and water retention by the kidney, and neurohumoral activation. Indeed, with increasing life expectancy, the incidence of HF is most likely to increase further. As such, it is imperative that improvements be made for the treatment of HF conditions. This will certainly help relieve the stress on utilization of public health resources.

There are various methods that can be adopted for the treatment of HF conditions. One of the ways is to administer exogenous natriuretic peptides (NPs) into HF patients. Several synthetic versions of naturally-occurring NPs have been developed and tested. These include Carperitide and Anartitide (synthetic Atrial-NPs) and Nesiritide (synthetic B-type NP) [2], [3], . Carperitide has been approved for treatment of HF patients in Japan since 1995 while Nesiritide was approved by FDA for the treatment of acutely decompensated heart failure in 2001. The use of synthetic NPs, which models the naturally-occurring variants, has its limitations. Although Atrial NP (ANP), B-type NP (BNP) and C-type NP (CNP) are structurally similar, they each exert properties that are physiologically distinct from one another. ANP and BNP, for instance, possess beneficial natriuretic and diuretic functions which are lacking in CNP. However, clinical usage of ANP and BNP is limited by their influence on renal perfusion pressure and arterial vasodilating properties. CNP, unlike the ANP and BNP, exhibits fibroblast-inhibition property which renders it particularly useful for cardiac protecting functions [7], [8], [9]. However, it lacks renal-protective functions such as natriuresis [10], [11] and diuresis. CNP also exhibits veno-dilating properties which makes it more attractive than the other NP variants in that it is less hypotensive [7], [8], [12].

Researchers at the Mayo Clinic have engineered a novel chimeric natriuretic peptide which is currently undergoing clinical testing. This novel NP, intuitively named as CD-NP, consists of 22-amino acid (AA) of CNP and 15-AA of a green-mamba derived peptide, called dendroapsis natriuretic peptide or DNP. CD-NP was synthesized with the goal of combining the complementary profiles of CNP and DNP into a single chimeric NP. It is shown that CD-NP which acts on both Natriuretic Peptide Receptors (NPR-A and NPR-B), can avoid the hypotensive nature of BNP which retaining the cardiac unloading properties and protective renal functions in congestive HF setting [13], [14], [15].

Presently, these peptides are administered intravenously while HF patients are hospitalized. However, despite the improvements in HF symptoms as a result of the treatment during hospitalization, there is still a high occurrence of re-admission and mortality rate associated with discharged patients. Currently, the only way to administer NP chronically, in a non-clinical setting, is subcutaneous (SQ) bolus administration of BNP. However, due to its short half life, multiple SQ injections have to be administered to ensure a constant therapeutic level of NPs in the circulatory system. This greatly reduces the patients' quality of life with increased risk of complications and is a potential source of reduced compliance. It is, therefore, imperative that some form of sustained drug delivery system is employed. This delivery system should protect the drug from enzymatic degradation while maintaining a sustained release of peptide drug over an extended duration.

The concept of an in situ polymer precipitation system (or gel) was first proposed by Dunn et al [16], [17], [18], [19]. It works on the principle of polymer precipitation due to water-solvent exchange. Solvents commonly accepted for used in in-situ forming drug delivery system include N-methyl-2-pyrrolidinone (NMP), dimethylsulfoxide (DMSO), 2-pyrrolidone, PEG400 and triacetin. NMP, which is used in FDA-approved Eligard® delivery system [20], [21], [22], is the most frequently used solvent in in-situ system due to its relative low systemic toxicity [23], [24], [25]. Triacetin, which is generally recognized as safe (GRAS) ingredient by FDA, has been reported to have a relatively low acute parental toxicity [26].

This delivery system has many advantages over other drug delivery systems. It is very easy to synthesize and it can be used to release different types of drugs i.e. peptide or protein molecules. Most importantly, administering of the gel is an easy process which the patient can carry out himself.

In this study, we report on our attempts to design an in situ polymer precipitation system that might be suitable for the release of the peptide drug. The precursor of this system involves a biodegradable and biocompatible polymer that can be dissolved in a biocompatible (usually organic) solvent. The drug is added into the mixture, forming a solution or suspension which can be easily injected into the body. When injected, interaction with the physiological environment results in the precipitation of the water-insoluble polymer. During the process of polymer precipitation, the drug will be encapsulated into the polymer matrix. Subsequently, this drug-containing polymer matrix acts as a depot at the injection site from which drug is released over an extended duration as the polymer degrades hydrolytically.

While CD-NP might have tremendous beneficial effects on both cardiac and renal functions, its efficiency is greatly reduced by its short 20 minutes in-vivo half-life [27]. Hence, the objective of this study is to investigate on the feasibility of using in situ polymer precipitation system for prolonged delivery of bioactive CD-NP.

## Materials and Methods

### 1. Materials

Poly (D, L-lactic-co-glycolic acid) (PLGA) (17 kDa, 50∶50 dl-LA to GA ratio, inherent viscosity: 0.2dL/g) from Purac Biomaterials, Gorinchem Netherlands, was used in the gel formulations. HPLC grade N-methyl-2-pyrrolidinone (NMP) and triacetin, purchased from Sigma Aldrich and fisher scientific respectively, were used as solvents. Both solvents are of low toxicity and are recommended for use in in-situ forming drug delivery systems. CD-NP peptide was supplied by Nile Therapeutics, Inc.

### 2. Synthesis of in situ polymer precipitation delivery system

Preparation of the injectable gel formulation is as followed: Briefly, CD-NP is dissolved in NMP solvent and the resulting suspension is allowed to stir for at least 3–4 hours before the addition of pre-weighed polymer and triacetin solution. The quantities added were such that the polymer and triacetin content was 40% and 20% by weight respectively, with the CDNP being present at 0.45% w/w. The final solution was allowed to homogenize overnight. Fresh gel samples were prepared 1 day in advance for each injection.

### 3. In-vitro studies

There are many factors contributing to the release profile of a gel system [18], [19]. Before in-vivo evaluation, several gel formations were tested in in-vitro. Effects of different gel parameters namely polymer concentration, single and co-solvent system, NMP/Triacetin concentration, CD-NP drug loading and gel injected volume were investigated. Special attention was also given to gel viscosity (affecting syringeability), initial burst of the gel system and lineage of the release profile within the first 4 weeks.

Briefly, 0.7cc of gel was injected into 10 ml of PBS buffer (pH 7.4) through a 20G needle. Sample vials were placed on a 3-ways rotator set at 100 rpm and incubated at 37°C. On each sampling day, the release medium was collected and replaced by 10 ml of fresh buffer. The collected release medium was tested for peptide concentration using microBCA Protein Assay Kit purchased from Thermo Scientific, Pierce Protein Research Products [28]. A final gel formulation that fulfilled the desired low initial burst and subsequent linear release profile was selected for in-vivo testing.

### 4. In-vivo studies

#### Animal care and preparation

The injectable gel study rat protocol was approved by Mayo Clinic Animal Care and Use Committee, Rochester, MN (Mayo IACUC Number: A34809). Isoflurane (1.5% in oxygen) was used to anesthetize the animal to minimize its suffering. Ventilation was provided using a rodent ventilator.

Male Wistar rats (Charles River Laboratories – Wilmington, MA), weight of 150–250 g, were used and randomly assigned into 4 groups of 5 rats each. Rat groupings as follows; Group 1–1 week gel treatment, Group 2–2 weeks gel treatment, Group 3–3 weeks gel treatment and Group 4– Vehicle group. All rats in this study were maintained on a standard laboratory diet and were initially allowed at least 3–4 days to acclimatize to the animal facility housing prior to the start of the study.

#### Gel implantation

In an effort of minimizing suffering on the animals during gel implantation, the rodents were anesthetized with isoflurane (1.5% in oxygen) and ventilation was provided using a rodent ventilator. To minimize any unnecessary risk of infection, the injection sites (back of the rat) were shaved and swapped with 70% ethanol prior to injection. The desired CD-NP dose was 10^−7^ g/kg/min and this was calculated based on a previous study [29]. 1.4cc of freshly prepared gel were drawn into a 3cc syringe and a 20G needle was then attached to the syringe tip. Approximately 0.2cc of gel was injected subcutaneously into each of the 7 sites on the back of the rodent. After the injection, the rodents were returned back to the cage and allowed to recover. A second injection, an exact procedural repeat of the first injection and on different sites, was carried out 48 hours later.

#### Blood pressure measurement

One of CD-NP properties for unloading of the heart is through veno-vasodilation. This results in a drop in blood pressure. As a non-invasive means of assessing the presence of circulating CD-NP released from the gel system, rodents' blood pressure was measured at pre-determined timepoints and monitored over an 18 days duration. Rodents' blood pressure was measured using the CODA Non-invasive Blood Pressure System for Mice and Rats from Kent Scientific Corporation.

#### Plasma evaluation

Treatment groups were sacrificed for blood collection at the respective sacrificial time points i.e. 1 week, 2 weeks and 3 weeks for plasma evaluation. Plasma analysis for the vehicle group was carried out at 3 weeks after injection. Radio-immumo Assay (RIA) Kit obtained from Perkin Elmer Life Science was used to evaluate plasma CD-NP and cGMP concentration [30]. The RIA assay works on competition between ^125^I-peptide and CD-NPs binding onto a limited quantity of antibodies.

#### Urinary output evaluation

24 hrs urine were collected at 4 respective time points namely baseline (before the start of study), after injection 1, after injection 2 and before sacrificial timepoints. During the 24 hrs urine collection, individual rodents are placed into individual metabolic cages where urine was collected. Urinary volume was noted and tested for the presence of urinary CD-NP and cGMP.

#### Statistics analysis

All data are expressed in mean ± SEM. The comparison between each measurement was performed by t-test. Significant difference of p<0.05 was accepted.

## Results and Discussion

### 1. In-vitro buffer release

**Figure 1:**
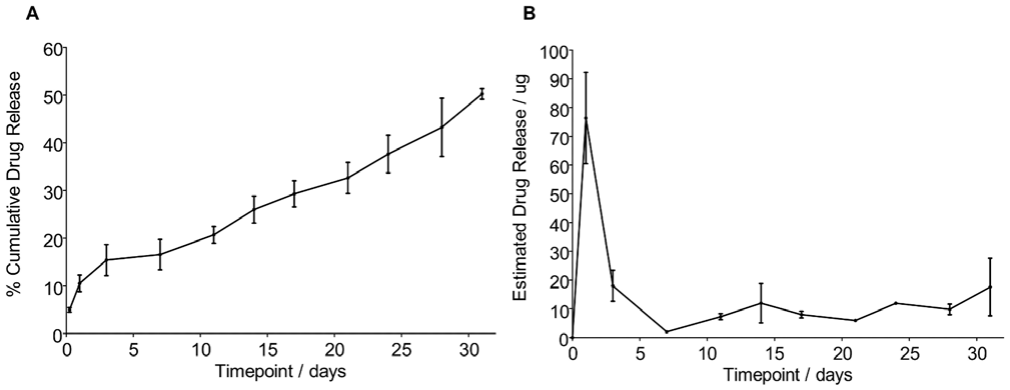
In-vitro Release of CD-NP over a 31 days durations from gel formulation. A) Cumulative Drug Release B) Estimated Daily Amount of Drug Release.

The nature of CD-NP peptide offers challenges for the gel formulation development. CD-NP is a short chain molecule and this makes controlling its release difficult. After investigating the effects of different parameters on the CD-NP release profile, a final gel formulation that mimics the desired low initial burst and subsequent zero order release was selected. The release profile for the selected gel formulation, presented in figure 1A, demonstrated a sustained CD-NP release for more than 30 days. It has an initial burst of 11±1% of the total CD-NP loading. Thereafter, rate of CD-NP release starts to decrease and assumes a more linear release over the next 30 days. Up to 50±1% of the total CD-NP loading was released by day 31.

Based on the CD-NP concentration data collected at each timepoint, an estimate of the amount of CD-NP released on a daily basis was generated. This is shown in figure 1B. Although the initial burst was relatively low, at 11±1%, this translates into 76±15.9 μg of CD-NP released. However, the burst effect was only observed within the first day. As soon as the “gel” is fully precipitated, CD-NP was released at a consistent level between 5.95 to 11.9 μg/day for the next 30 days.

The targeted dose of 10^−7^ g/kg/min was based on a C-NP study by Soeki et al. on its effect in a myocardial infarction model [29]. In order to achieve this dosage in the rat, the amount of gel required was quadrupled from 0.7cc used in in-vitro to 2.8cc. Bearing in mind the effects of the initial burst of the gel system, precaution was taken by separating the required 2.8cc into 2×1.4cc injections. Injections were carried out on Day 0 and on Day 2. This allows a 48 hours recovery window in which the initial burst effect would dissipate.

Release profile of any injected gel is dependent on the kinetics of phase inversion as well on the rate of polymer matrix degradation. This, in turn, is dependent on intrinsic and extrinsic factors, one of which is the availability of water as the polymer matrix degrades hydrolytically. Studies have shown that in the subcutaneous environment, the relatively limited availability of water means that the polymer matrix tends to degrade at a slower rate as compared to that in vitro [31]. As such, instead of injecting the entire 1.4cc of gel into a single site, a total of 7 multiple sites were injected with approximately 0.2cc of gel each. It was hoped that given the larger surface area of the smaller gel, the polymer will degrade faster and hence achieve the desired release profile that most resembles the in-vitro data.

### 2. In-vivo studies

With one exception, all rats recovered well after each gel injection. Recovery typically takes place within hours after injection. The death of the single rat, from the vehicle group, occurred after the second gel injection. Cause of death could be due to the series of events that the rat experienced following the first injection. Rats undergo multi-anesthetization on Day 0 and 2 during gel injection. In between the injections, the rats are subjected to multiple blood pressure measurements. Procedure for blood pressure measurement involves holding the rats in a confined space that is heated to 37°C. This series of events could be too traumatic for the rat.

#### Blood pressure measurement

**Figure 2:**
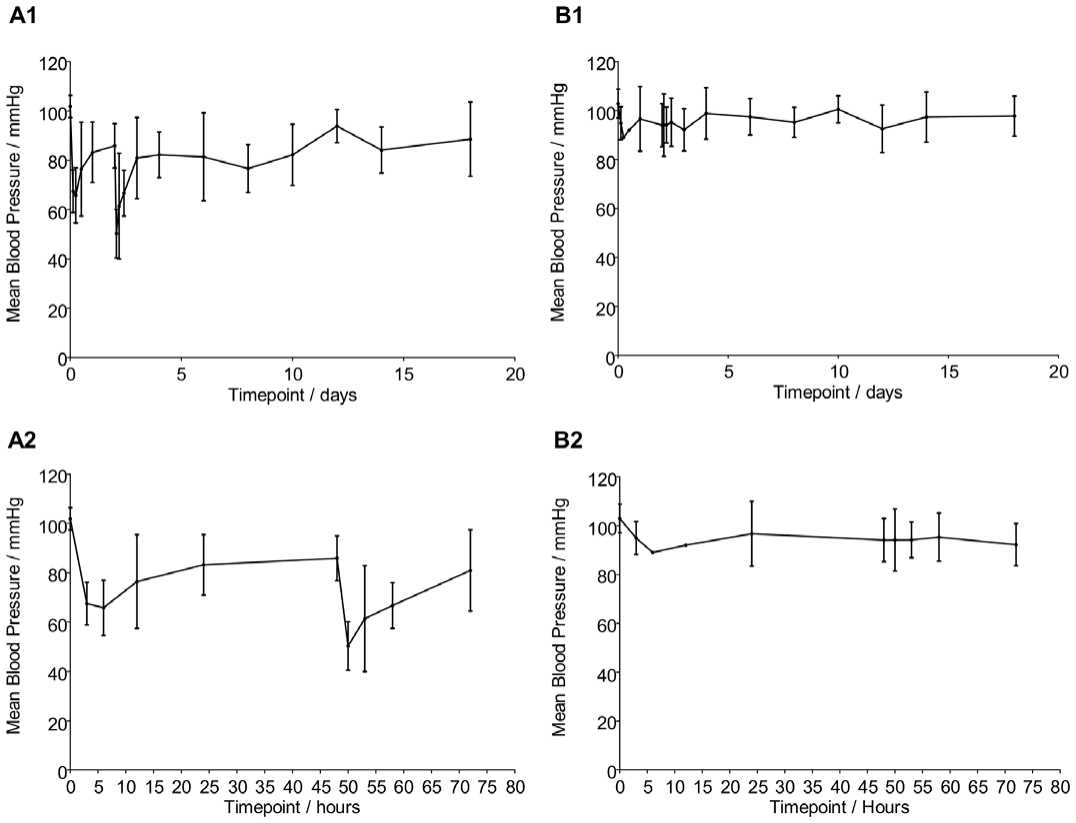
Blood Pressure Fluctuation over 18 days duration. A1) BP trend for CD-NP treatment groups A2) Magnified 3 days BP trend for treatment group B1) BP trend for vehicle group B2) Magnified 3 days BP trend for vehicle group. All timepoint is significantly different except for 0d and 12d, Compared between BP of treatment and vehicle group of the same timepoint.

As a non-invasive way of assessing the presence of circulating CD-NP levels in the rodents, blood pressure was measured. Given the veno-dilating property of CD-NP, any presence of circulating CD-NP will be indicated by a lower blood pressure. As such, the blood pressure (BP) was monitored for both the treatment and vehicle group. BP measurements were taken once every 2–3 days with more readings taken within 24 hrs after each injection. Figure 2– A1 and B1 shows the mean BP trend measured over 18 days of the study for the treatment and vehicle group respectively. Figure 2– A2 and B2 shows the mean BP trend, magnified over the initial 3 days.

BP trend for the vehicle group shows a more consistent mean BP of between 89.0 to 100.6 mmHg throughout the study duration. There are however, some drops in BP observed during the first 3 days of the study which is mostly likely due to the repeated exposure to isoflurane during the anesthetization process.

As expected, the mean BP for the CDNP-treated groups is seen to have dropped from 102±1.2 mmHg to 66±1.7 mmHg within the 1st 6 hrs after gel injection 1. This drop coincides with the initial burst release from the gel system. The BP recovers to approximately 83±1.8 mmHg within the 1st 24 hours. Following the 2^nd^ injection on Day 2, the BP dropped from 86±1.1 mmHg to 50±3.0 mmHg. After the BP recovery from the initial burst effect of the 2^nd^ injection, the mean BP was maintained between 76.7 mmHg to 90.9 mmHg until the end of the 18^th^ day. This lowered BP is significant as compared to the vehicle. The BP trend from the CDNP-treated group is confirmation that CD-NP is constantly being released from the gel system over the entire 18 days test duration, and is exerting its bioactive function of vaso-dilation. CD-NP infusion study by Lisy et al. on normal dogs has also demonstrated the lowering of mean arterial pressure and that the change in BP is CD-NP dose dependent [15].

#### Plasma concentrations

**Figure 3:**
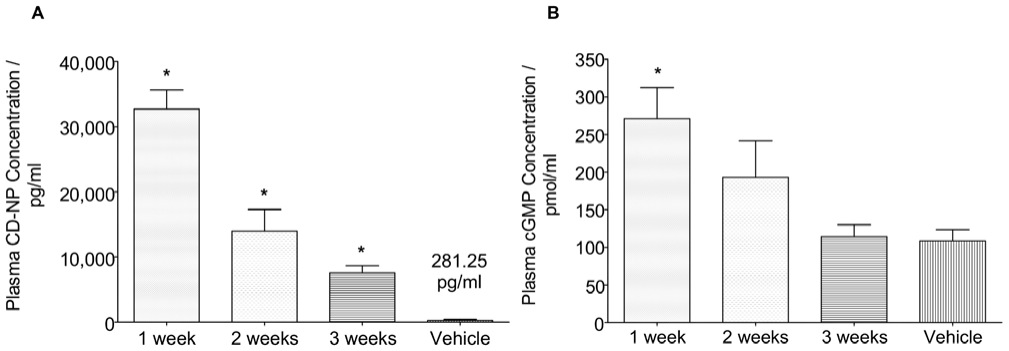
Plasma Concentration Analysis . A) Plasma CD-NP concentration at 1/2/3 week timepoint B) Plasma cGMP concentration at 1/2/3 week timepoint *P<0.05, Treatment vs Vehicle.

Plasma CD-NP concentration (pCD-NP conc.) was quantified using the Radio-immumo Assay (RIA) Kit. Shown in figure 3A, results of the pCD-NP conc. demonstrated a sustained release of CD-NP over the 3 weeks study duration. Measured pCD-NP conc. was 33,000±2888 pg/ml, 14,000±3302 pg/ml and 8,000±1115 pg/ml at 1/2/3 weeks respectively. pCD-NP conc. measured from the vehicle group is found to be around 280±160.4 pg/ml. The results from the CDNP-treated groups clearly demonstrated an elevated pCD-NP level that is significantly different from the vehicle group.

If the in-vitro data is representative of in vivo release, one would expect the pCD-NP conc. for the week 3 timepoint to be similar to that of week 2. However, this is not the case. There are two possibilities for this discrepancy. Firstly, it could be due to the difference in rate of polymeric degradation of the polymer matrix in vivo as compared to in vitro, and secondly, a possible loss of CD-NP bioactivity. PLGA polymer degrades through hydrolysis which is highly dependent on pH and the availability of water. In the in-vitro setting, the injected gels are surrounded by buffer medium which are constantly being replaced with fresh buffer during each sampling timepoint. On the other hand, there is generally less availability of water in the subcutaneous tissue for uptake into the injected gel. As such, the degradation rate for the in-vivo gels might be relatively slower. This may account for the slower release and hence the lowered CD-NP release over the 3 timepoints. This reduction in degradation rate between in vitro and in vivo was also observed in other studies [31]. Another possible explanation for the decrease in measured pCD-NP could be due to the degradation of CD-NP. As PLGA matrix degrades into lactic and glycolic acids, it is will create pockets of slightly acidic environment in the polymer matrix [31]. This internal acidic environment might degrade the fragile CD-NP peptide. Since the RIA kit only works well in detecting intact CD-NP, the resulting pCD-NP conc. could be lower than expected.

To test for the bioactivity of the released CD-NP, plasma Cyclic Guanosine 3′5′Monophosphate concentration (pcGMP conc.) was measured. cGMP is a secondary messenger activated through the binding action of CD-NP onto NPR-A/B receptors. It has been shown that only an intact ring structure will activate NPR-A receptor [32], [33]. Hence, the presence of cGMP will certainly confirm the biological activity of released CD-NP. From the pcGMP conc. results shown in figure 3B, the pcGMP conc. was measured to be 270±41.4 pmol/ml, 190±48.1 pmol/ml and 114±15.8 pmol/ml at the respective 1/2/3 weeks timepoints. pcGMP conc. from the vehicle group shows a conc. of about 108±15.5 pmol/ml. Although pcGMP conc. was significantly elevated at week 1 as compared to vehicle, there was a less significant difference for week 2 and 3 measurements when compared to the vehicle.

The plasma cGMP concentration is dependent on the amount of pCD-NP and availability of NPR-A/B receptors. Studies have demonstrated that CD-NP activation of cGMP is dose-dependent and this relationship plateaus at high CD-NP conc. [15], [32]. Based on unpublished data from previous studies by Burnett et al, it was determined that the maximum pcGMP conc. in this particular rodent species to be between 250 pmol/ml to 300 pmol/ml. Given the high level of pCD-NP administered, one would expect maximum activation of cGMP throughout the 3 weeks duration. However, this was not observed.

There is one possible explanation for this observation. In this pilot study, the objective is to achieve a sustained release effect of CD-NP from the gel system. As such, an excess of CD-NP was loaded into the depot. This meant that, as discussed in the previous section, a high level of pCD-NP in plasma was achieved even at 3 weeks. The abundant availability of pCD-NP, led to continual maximum activation of cGMP over an extended duration. This high cGMP activation rate might not be perceived by the body as ‘normal’. As such, compensatory responses might intervene to regulate this abnormally high amount of pcGMP. There are several possible compensatory responses. Within the natriuretic peptide pathway, there might be a partial down regulation of NPR-A/B receptors. This prevents further binding of CD-NP onto the receptor. NPR-C receptors, also known as the NPs clearance receptor, could go into over-drive, increasing the degradation of CD-NP. These responses will reduce the rate of cGMP activation. A second mechanism for the decreased in plasma cGMP may be in part due to increased cGMP excretion via the kidneys as supported by the increased urinary cGMP excretion. A third mechanism may be due to the degradation of cGMP by phosphodiesterases (PDE) such as PDE V. This will be discussed further in the following section.

#### Urinary output evaluation

Table 1 shows the 24 hrs urinary output for both the treated and vehicle rodent. Mean 24 hrs baseline urine output for the treated and vehicle group is 15±1.3 ml and 13±0.9 ml respectively. After the first and second gel injection for the treatment group, the mean urinary output increased to 32±2.5 ml and 32±1.8 ml respectively. The increase in urinary volume output is approximately twice that of the baseline volume. CD-NP is also known to be diuretic [15], [32]. As such, the increase in the urinary output right after the injections is related to the amount of CD-NP administered during the 1st 24 hours after each injection. This is further evidence suggesting that CD-NP released from the gel polymer matrix is biologically active.

**Table 1:** 24 hours Urinary Output Volume for Baseline, 1^st^ & 2^nd^ post-injection and prior to sacrificial.

Timepoint	TreatGroup (ml)	Vehicle Group (ml)
Baseline	15 ed	13
After Injection 1	32.4	-
After injection 2	31.5	-
1 week	11.9	-
2 weeks	14	-
3 weeks	12.5	11.9

**24 hrs Urine Collection Volume.**

**Figure 4:**
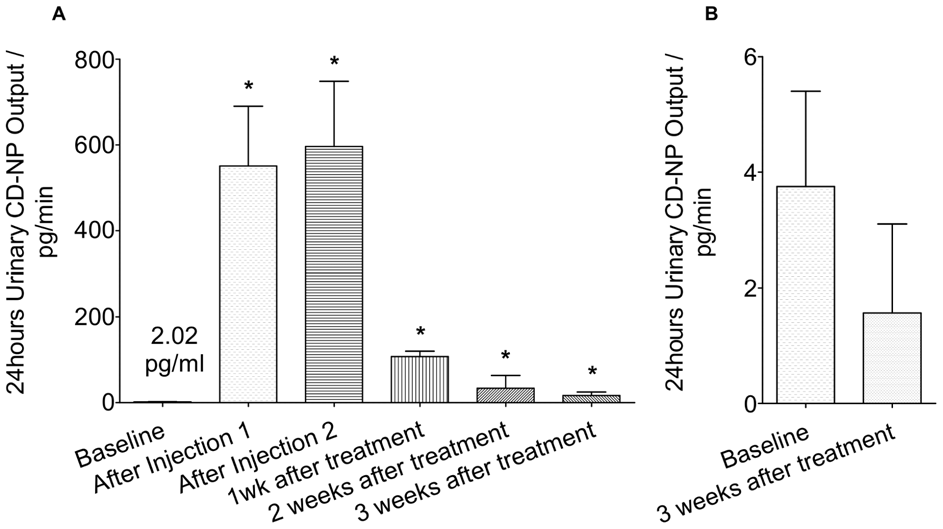
24 hours Urinary CD-NP Output. A) Treatment Groups B) Vehicle Group *P<0.05, Treatment vs Vehicle.

Background urinary CD-NP (uCD-NP) level, as shown in figure 4, is measured at 2±0.10 pg/min and 4±1.65 pg/min for treatment and vehicle groups respectively. For the treatment group, the uCD-NP was measured to be at 550±139.60 pg/min and 600±151.90 pg/min after the 1^st^ and 2^nd^ injection respectively. Immediately after the gel injections, there is a burst release of CD-NP from the gel. Most of this un-used CD-NP will be excreted out of the system through urinary excretion. This accounts for the high uCD-NP output. The amount of CD-NP excreted at 1/2/3 weeks reduced to 107±12.70 pg/min, 34±29.72 pg/min and 17±8.19 pg/min respectively. These are statistically significant when compared with the baseline level. As mentioned, following the burst release, the amount of CD-NP released from the gel system will reduce to a more sustainable level. This, in turns, led to the lower uCD-NP output observed. There is no significant difference in uCD-NP output observed between the baseline and 3 weeks urinary output for the vehicle group.

**Figure 5:**
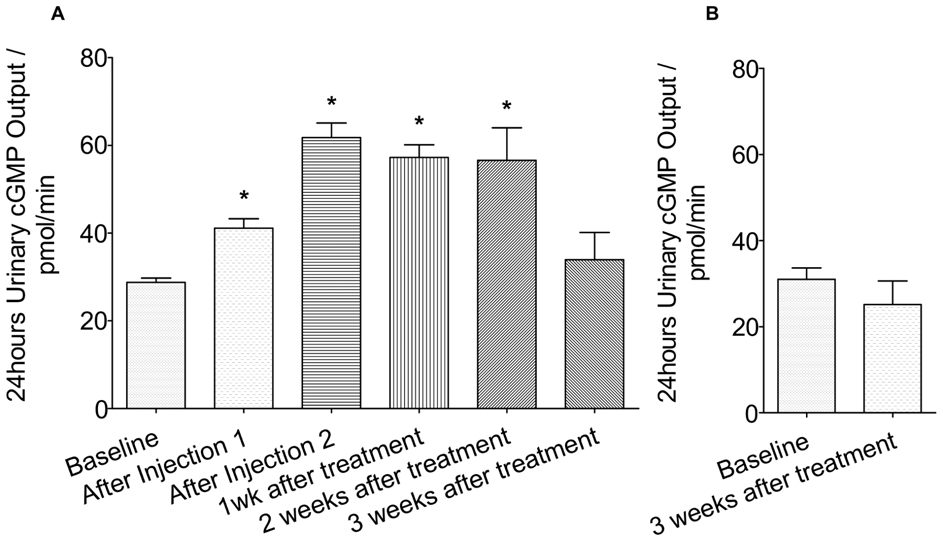
24 hours Urinary cGMP Output. A) Treatment Groups B) Vehicle Group *P<0.05, Treatment vs Vehicle.

Shown in figure 5, baseline urinary cGMP (ucGMP) level is measured at 29±0.10 pmol/min and 31±2.68 pmol/min for treatment and vehicle group respectively. There is no significant change in the ucGMP output for the vehicle group at 3 weeks after the injection of the blank gel. As for the treatment groups, ucGMP output was significantly increased following gel injections. ucGMP output was 41±2.16 pmol/min and 62±3.32 pmol/min after the 1st and 2nd injection respectively. The ucGMP output after the 1st injection was slightly lower than that measured for the 2nd injection. This is probably due to the fact that there is a lapse time between CD-NP diffusing out of the gel system into the blood, binding of CD-NP with NPR-A/B receptors and the activation of cGMP to the filtration of pcGMP in the kidney. ucGMP output continued to remain high at 1 and 2 weeks. It has, however, dropped to a level that is not significant at 3 weeks after the treatment. One interesting observation is that the ucGMP output level was consistently high at week 1 and 2.

In the in vivo results and discussion section, we reported observations from blood pressure, plasma CD-NP & cGMP and urinary CD-NP & cGMP. We have demonstrated an elevated pCD-NP level throughout the 3 weeks treatment duration. The high level of recorded uCD-NP is consistent with the elevated pCD-NP. Bioactivity of released CD-NP was also proven to be retained, as seen by the initial elevation of pcGMP level at week 1, and by the sustained hypotensive state of the treated rats.

However, over longer times, pcGMP level did not sustain the saturation level that was predicted by the level of pCDNP. Following pcGMP saturation level at week 1, pcGMP gradually decreased over the next 2 weeks to a level that is not significantly different from the baseline level. Evidence from ucGMP excretion suggests that this decreased in pcGMP is not due to the loss of bioactivity of released CD-NP. Despite the decreasing pcGMP level from week 1 to week 3, a constant high level of ucGMP was recorded up to 2 weeks into treatment. Instead, we believe that following the initial days of saturation level of cGMP production, the body may have perceived the level as ‘abnormal’. As such, compensatory responses may intervene to regulate this abnormally high level of circulating pcGMP. The compensatory responses are of two types, both of which will help reduce the circulating pcGMP.

The first mechanism works by reducing the high cGMP activation rate. Within the NP/cGMP pathway, there could be down regulation of NPR-A/B receptors thus reducing cGMP production. NPR-C receptor might also be ‘set’ into over-drive mode to rapidly remove circulating CD-NP. This will aid in reducing the pCD-NP concentration and in turn, will reduce the availability of CD-NP for binding onto NPR-A/B receptors. This increased pCD-NP clearance is also confirmed by the elevated uCD-NP output. Outside of the NP/cGMP pathway, there is another cGMP pathway known as NO-sGC/cGMP pathway. This pathway also promotes cGMP production and could also be down regulated to further reduce cGMP production.

The second mechanism is aimed at reducing the circulating pcGMP level. This could be achieved by increasing cGMP removal rate through degradation of cGMP by phosphodiesterases or more directly through urinary excretion.

Based on the high level of sustained ucGMP output that was recorded, we propose that the dominant reason for the gradual drop in pcGMP level from week 1 to week 3 is due to the high removal rate via urinary excretion.

## Conclusion

The use of in situ polymer precipitation delivery system is certainly an attractive approach for the delivery of the novel chimeric CD-NP in improving patient compliance and quality of life. This study demonstrated that with the appropriate gel formulation, CD-NP release could be sustained over a 3 weeks duration. Bioactivity of released CD-NP is also maintained up to 3 weeks although not at the levels expected from in vitro data. This pilot study also provides insight that with a CD-NP delivery system, it might not be necessary to sustain a high level of CD-NP release. The key is to determine the right dosage of CD-NP that is needed to stimulate a slightly elevated but not over-excessive amount of cGMP. This delivery of CD-NP through subcutaneous gel implantation is certainly a viable way. Given an appropriate gel formulation and drug loading, this mode of delivery will certainly be beneficial to heart failure patient.

### Limitations of this study

Findings from this pilot injectable gel study demonstrated that it is critical to further minimize the initial burst effect of the gel system. Marker of bioactivity of released CD-NP was based on cGMP level. With antagonistic system coming into influence in in-vivo setting, it might be necessary to test for other biological markers to further assert the actual influence of such system. Possibly, gel system should also be used in diseased models to potentiate the efficacy of the system.
